# Popular Study of Anatomy and Physiology

**Published:** 1834

**Authors:** 


### II. Popular Study of Anatomy and Physiology.

The Editor after having been repeatedly urged by a number of respectable teachers, of both sexes, has commenced the composition of a school and college book of *Anatomy*, *Physiology and Hygeine*. It will run through about 300 duodecimo pages, and be illustrated with numerous engravings on wood.

The organs and functions which are not of general interest, and those which cannot, with propriety, be introduced into a work designed for all kinds of readers, will be omitted, together with all surperfluous technical terms.

Having stated what the work will *not*, it may be well to say something of what it *will* contain.

An account of the skeleton, with observations on its mechanism, and deformities—muscular motion, with the influence of exercise—the brain, with an accout of the science of phrenology—the distribution and classification of the nerves—connexion between the nerves and the muscles—the senses—the intellectual functions, with an account of the relation of mind and body on each other, and the connexion of physiology with mental physiology—the heart and circulation of the blood—action of the blood upon the brain—the lungs and respiration—connexion between the functions of circulation and respiration—the stomach and liver—digestion—bilious secretion—the secretions generally—influence of the digestive functions over those of the brain and *vice versa*—effects of intemperance in eating and drinking—diseases which are the offspring of civilization—pernicious effects of premature or excessive study—of excited passions—of sedentary habits and occupations and of confined air, &c. &c.

The Editor wishes, in this notice, to make an appeal to the profession in favor of his undertaking. Believing that Anatomy and Physiology, to a certain extent, should be made a study in every literary institution which teaches Chemistry and Natural Philosophy, but aware, that not a little of popular prejudice must be encountered, he would, respectfully, solicit the aid of his professional brethren. They may do much to promote the introduction of this study into our higher schools and colleges; and they are deeply interested in making it a branch of general education. It is now a universal complaint, in the profession, that the people do not distinguish between its learned and its ignorant members. This they cannot do, from the want of knowledge themselves, but supply that want, make them acquainted with the first principles of Anatomy and Physiology, and they will soon come to discriminate between the man of science and the empyric. But to take a less selfish view of this matter, it may be said, that if it would be beneficial to society, to have Anatomy and Physiology made a branch of liberal education, physicians should interest themselves in the matter, because their efforts would have greater effect, than those of any other class of men.

In the next number of the Journal, the Editor proposes to give a specimen of the work, that his brethren may judge for themselves, of its adaptation to the end in view.

